# Current Treatments to Control African Trypanosomiasis and One Health Perspective

**DOI:** 10.3390/microorganisms10071298

**Published:** 2022-06-27

**Authors:** Alberto Venturelli, Lorenzo Tagliazucchi, Clara Lima, Federica Venuti, Giulia Malpezzi, George E. Magoulas, Nuno Santarem, Theodora Calogeropoulou, Anabela Cordeiro-da-Silva, Maria Paola Costi

**Affiliations:** 1 Department of Life Sciences, University of Modena and Reggio Emilia, 41125 Modena, Italy; alberto.venturelli@unimore.it (A.V.); lorenzo.tagliazucchi@unimore.it (L.T.); federica.venuti93@gmail.com (F.V.); giulia.malpezzi@gmail.com (G.M.); 2 Doctorate School in Clinical and Experimental Medicine (CEM), University of Modena and Reggio Emilia, 41125 Modena, Italy; 3 Host-Parasite Interactions Group, Institute of Research and Innovation in Health, University of Porto, 4099-002 Porto, Portugal; clara.lima@ibmc.up.pt (C.L.); santarem@ibmc.up.pt (N.S.); cordeiro@ibmc.up.pt (A.C.-d.-S.); 4 Department of Biological Sciences, Faculty of Pharmacy, University of Porto, 4099-002 Porto, Portugal; 5 Institute of Chemical Biology, National Hellenic Research Foundation, 11635 Athens, Greece; gmagoulas@eie.gr (G.E.M.); tcalog@eie.gr (T.C.)

**Keywords:** human African trypanosomiasis, animal African trypanosomiasis, antitrypanosomal drugs, drugs mechanism of action, one health approaches

## Abstract

Human African Trypanosomiasis (HAT, sleeping sickness) and Animal African Trypanosomiasis (AAT) are neglected tropical diseases generally caused by the same etiological agent, *Trypanosoma brucei*. Despite important advances in the reduction or disappearance of HAT cases, AAT represents a risky reservoir of the infections. There is a strong need to control AAT, as is claimed by the European Commission in a recent document on the reservation of antimicrobials for human use. Control of AAT is considered part of the One Health approach established by the FAO program against African Trypanosomiasis. Under the umbrella of the One Health concepts, in this work, by analyzing the pharmacological properties of the therapeutic options against *Trypanosoma brucei* spp., we underline the need for clearer and more defined guidelines in the employment of drugs designed for HAT and AAT. Essential requirements are addressed to meet the challenge of drug use and drug resistance development. This approach shall avoid inter-species cross-resistance phenomena and retain drugs therapeutic activity.

## 1. Epidemiology of African Trypanosomiasis

**Figure 1:**
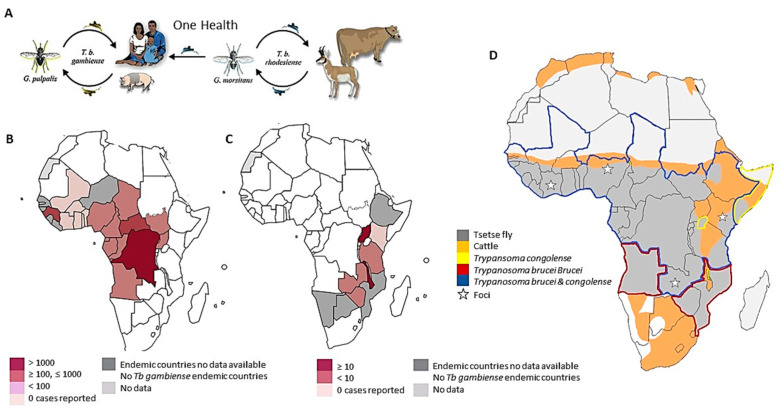
The transmission cycle and distribution of HAT (**A**) caused by *T. b. gambiense* has humans and domestic animals, especially pigs, as the main hosts. It is transmitted by the tsetse flies (*Glossina *sp.) of *G. palpalis* group and is distributed in Western and Central Africa (**A**,**B**). HAT caused by *T. b. rhodesiense* has wild animals and cattle as the main hosts. It is transmitted by *G. morsitans* and is distributed in Eastern and Southern Africa (**A**,**C**) [1]. (**D**). The map shows the distribution of the tsetse fly and cattle, AAT caused by *T. congolense,* and HAT caused by *T. b. brucei*. Each area of interest is within a colored line or with colored content as reported in the legend. The transmission between humans and animals causes a One Health problem (**A**,**D**). (Figure 1A,B were adapted with permission from references [1]. Copyright year 2022, Elsevier under the license number 5314910954512, 23 May 2022).

Trypanosomiases are clinical infections caused by the hemoflagellate Trypanosome protozoan parasites, occurring as both veterinary and public health problems in sub-Saharan countries. Two different epidemiological contexts meet at a zoonotic interface powered by tsetse flies: Animal African Trypanosomiasis (AAT), with implications for both livestock and wildlife, and Human African Trypanosomiasis (HAT), also known as “sleeping sickness”. HAT is caused by the etiological agent *Trypanosoma brucei (T. b.*) *gambiense*, present in Western and Central African countries, and the zoonotic *T. b. rhodesiense*, found in over 13 countries of Eastern and Southern Africa regions (Figure 1). AAT is caused by different trypanosomes, including *T. b. rhodesiense.*

### 1.1. Human African Trypanosomiasis

The risk of Trypanosomiasis and of an upsurge of sleeping sickness is strictly related to impoverishment, migration, draught, armed conflicts, deforestation, and forcing exposure of humans and livestock to tsetse flies (*Glossina* sp.) in tsetse-inhabited areas. On the other hand, socio-economic instability, and geopolitical disputes compromise the fragile disease surveillance systems [2]. Currently, the emergency of HAT in sub-Saharan Africa is exposing 60 million people to the risk of infection. At the turn of the century, sleeping sickness reached epidemic proportions in Angola, the Democratic Republic of Congo, Uganda, and Sudan, and its prevalence has increased in Cameroon, Congo, Côte d’Ivoire, Central African Republic, Guinea, Mozambique, Tanzania, and Chad [3]. Notwithstanding, since 2000, when the Pan-Africa Tsetse and Trypanosomiasis Eradication Campaign (PATTEC) was endorsed, and WHO launched an initiative to reinforce the control and surveillance of HAT in 2001, the burden of *T. b. gambiense*-induced human chronic illness in Western and Central African fell by 98% (from 27,862 in 1998 to 565 in 2022) and the number of newly reported cases of the acute form HAT caused by *T. b. rhodesiense* fell by 84% (from 619 to 98) [4]. However, the disease remains sporadically observed in both local populations and travelers [4].

Humans represent the most important reservoirs of *T. b. gambiense*, while zoonotic *T. b. rhodesiense* is hosted by a wide range of domestic and wildlife species, including ungulates from Bovinae (e.g., African buffalo, *Syncerus caffer*) and Suidae subfamilies (e.g., warthog, *Phacocoerus africanus*), wild major carnivores (e.g., *Panthera leo*), and equids (e.g., bushbucks, *Tragelaphus scriptus*; impala, *Aepyceros melampus*; zebra, *Equus quagga boehma*; duiker; *Sylvicapra grimmia*). The presence of an animal reservoir of *T. b. rhodesiense* in regions where tsetse flies have been spreading uncontrolledly justifies the long-term endemicity of the most grandstanding HAT foci [5]. Nevertheless, human–animal proximity increases the risk of spill over and crossing species barriers, allowing the interchange of *Trypanosoma* spp. between animals and humans.

### 1.2. Animal African Trypanosomiasis

In 1898, Bruce identified *T. brucei* in cattle and later, in 1909, identified tsetse flies as the trypanosome transmitting vector [6]. Since then, several pathogenic *Trypanosoma* spp., have been associated with diseases of veterinary and public health impact generically known as Animal Trypanosomiasis (AT). In South America, AT is caused by zoonotic *T. cruzi*, *T. vivax* and *T. b. evansi* (agent of “surra”). In Asia, AT is caused by *T. evansi* [7]. African Animal Trypanosomiasis (also known as “nagana”) is endemic in at least 37 African countries. It is caused by a wider range of *Trypanosoma* spp., including *T. vivax* (domestic and wild ruminants, horses), *T. congolense* (in most domestic and many wild animals)*, T. simiae* (domestic and wild pigs), *T. b. brucei* (ungulates, dogs, cats, camels), *T. equiperdum* (equines), and *T. b. evansi* (camels, equines) [5,8]. Since indigenous African animal breeds have co-evolved with *Trypanosome* spp., the impact of trypanosome infection in wildlife animals and autochthonous African livestock breeds (e.g., N’dama cattle, Djallonke sheep and West African Dwarf goats) is balanced by trypanotolerance, the immune tolerance mechanism against *Trypanosome* spp. infection. The trypanotolerance has likely developed in response to natural selection pressures, which confer the capacity of infected animals to control parasite replication and limit their pathological effects, while trypanosomes preserve their ability to evade the host immune mechanisms [5,8]. Despite the lack of homogenous data and many difficulties in AAT detection and disease monitoring within the African regions, when available, current AAT and respective infecting species distribution maps suggest that these occur within the same areas where HAT is endemic, thus supporting the concept of AAT as a reservoir of HAT (Figure 1).

## 2. The Disease

### 2.1. The Human Disease

*T. b. gambiense* is responsible for a gradually progressive disease, accounting for 85% of reported cases of HAT in 2020. *T. b. rhodesiense* causes an acute fast-progressing form of sleeping sickness, representing 15% of reported cases [9,10]. Significantly, under-reporting and sub-notification together undermine the case report system of the World Health Organization (WHO) Global Health Observatory [11].

Humans living in HAT endemic areas have developed protective immune mechanisms against certain *Trypanosome* spp. otherwise pathogenic for animals (e.g., *T. b. brucei*). These include serum-specific trypanolytic factors, such as proteins responsible for determining the lysis of the parasite outer membranes. However, the two sub-species infecting humans, *T. b. gambiense* and *T. b. rhodesiense*, have evolved mechanisms to overcome these lytic resistance factors, making *T. b.*-infected hosts susceptible to disease. Noteworthy, the surface membrane of trypanosome parasites is coated with immunodominant variant surface glycoproteins (VSGs) [9,12]. During mammalian host infection, VSGs are recognized by the host’s immune system that upon production of antibodies, can neutralize and clear certain trypanosome species. Therefore, during infection, a constant low-frequency gene conversion process occurs switching VSG genes, resulting in a continuous process of antigenic variation. This enables the pathogenic trypanosomes to constantly evade the host’s immune responses, which would otherwise destroy the parasite. This phenomenon of antigenic variation is exacerbated by intraspecies variations in VSG genes and explains why vaccination against the trypanosome infection has so far been unachievable [12].

Complex host–parasite interaction mechanisms evolved to a clinical presentation of disease that comprises two stages, classically described as: (i) an initial hemolymphatic stage, characterized by parasite multiplication and dissemination from the blood and lymphatic system to the spleen, liver, heart, endocrine organs, and ophthalmic bulbs. No pathognomonic signs of HAT can be observed at this stage, except for Winterbottom’s sign, a posterior cervical region lymphadenopathy associated with *T. b. gambiense* infection. Intermittent fever, weakness, arthralgia, lymphadenopathy, pruritus, headache, lassitude, hemolytic anemia, hepatomegaly, and pericardial and myocardial involvement are more often described. Symptoms may be preceded by the development of a primary skin lesion detected at the site of *T. b. rhodesiense* inoculation (rarely found in *T. b. gambiense* infection) [13]. (ii) The second stage of disease starts with meningoencephalitis produced by the invasion of trypanosomes across the blood–brain barrier (BBB), affecting the central nervous system (CNS) and producing neurological signs, including: headache, myelopathy, myositis, cerebellar ataxia, tremors, and characteristic sleeping disorders (daytime somnolence, nocturnal insomnia, and sudden urges to sleep) [13]. Severity of clinical signs and speed of progression is linked to the parasite’s genetic variability and worsen for *T. b. rhodesiense*, for whom compartmentalized geographical distribution has produced genetically distinct parasite variants associated with disease features. However, most signs and symptoms are common to both stages, making it difficult to distinguish the two by clinical features alone [9]. If untreated, the patient will deteriorate with progressive impairment of consciousness, incontinence, seizures, and eventually death in most cases [13].

The development of acute or chronic disease depends on the infecting *T. brucei* subspecies, host response, and disease stage. Acute symptoms occur in *T. b. rhodesiense* infection, progressing to the second disease stage within a few weeks, and death may occur within 6 months. Chronic progressive disease is caused by *T. b. gambiense* infection, where sleeping sickness has a mean duration estimated at three years [14].

### 2.2. The Animal Diseases

The impact of AAT in livestock depends on the pathogenicity of the infecting *Trypanosome* sp. and the targeted animal species and breed. Overall, AAT may result in a chronic progressive disease manifested by weakness, inappetence, and dehydration, resulting in weight loss, lymphadenopathy, hemolytic anemia, leukopenia, thrombocytopenia, and potentially death. Degenerative and inflammatory lesions are observed in most organs during the later stage of the infection [5]. Particularly, acute hemorrhagic syndrome, besides lymphadenopathy and anemia, has been associated with *T. vivax* infection in cattle and horses. Pigs infected with *T. simiae* may suffer from neurological signs, besides anemia and weight loss. The most pathogenic trypanosome for livestock is *T. congolense*, which contributes to high morbidity and mortality rates among cattle. The risk of contagion is higher among people and communities living in the husbandry–wildlife boundaries [9]. If domestic cattle are infected, then they will be unusable for food and milk production, with significant socio-economic consequences [9]. Reduced productivity has been estimated to occur in over 150 million cattle and 260 million sheep and goats, which makes AAT responsible for direct and indirect agricultural production losses in Africa [10]. Approximately 35 million doses of trypanocidal drugs are administered annually. The annual losses in terms of cattle production alone exceed USD 1 billion, while the total direct and indirect economic losses in terms of agricultural Gross Domestic Product (GDP) are estimated at over USD 4 billion per year (https://www.fao.org/3/ca3887en/ca3887en.pdf, accessed on 12 April 2022).

## 3. Current Treatments and Therapeutic Challenges for HAT

**Figure 2:**
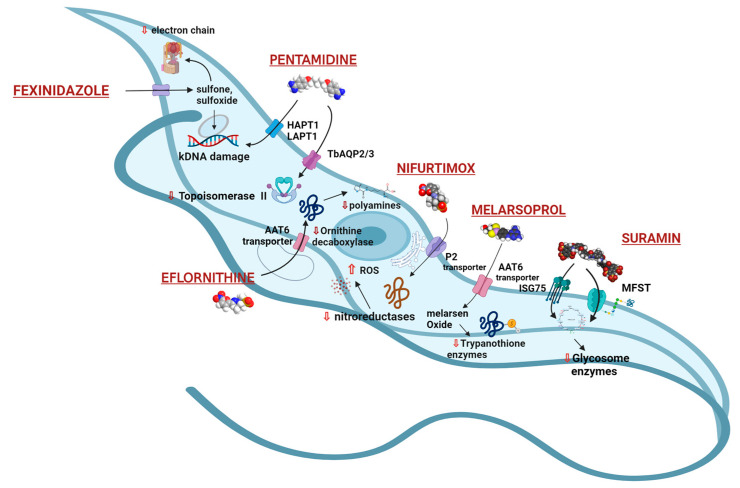

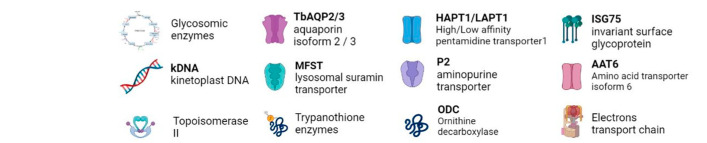
Drugs used to treat HAT and their mechanisms of action are illustrated. The different targets and transporters have been reported [41,42,43,44,45,46,47]. Created with Biorender.com, agreement number PT23Y61FRT 22 May 2022.

The weak worldwide interest for the development of new therapeutic options for the management of HAT led to the classification of Trypanosomiasis as a Neglected Zoonotic Disease (NZD) by the U.S. Department of Health and Human Services through the Centers for Disease Control and Prevention (CDC) and the WHO [15]. Moreover, since African Trypanosomiasis affects both animals and humans, and the therapeutic resources available for treatment are limited, the European Medicine Agency (EMA) anticipates that compounds from the same classes may be used to treat animals and humans [16]. The same document, however, suggests that a careful evaluation of drug resistance cases, if occurring, should lead to reservation of drugs for the exclusive treatment of HAT. Drugs approved for treating HAT are depicted in Table 1, and their targets represented in Figure 2. Both pentamidine and suramin are used during the first (hemolymphatic) stage. Melarsoprol and eflornithine are recommended for the second stage, since this involves neuro-migration of the parasites to the CNS. The major challenges associated with the available drugs include poor oral bioavailability (often intramuscular, IM, or intravenous, IV), toxicity, lack of efficacy, prolonged treatment leading to non-compliance, and sometimes, high costs [5,17].

### 3.1. Pentamidine and Suramin

Pentamidine (Table 1) is a diamidine bearing two positive charges at physiological pH. For early-stage infections caused by *T. b. gambiense,* drug therapy consists of IM (preferable) or IV pentamidine. Pentamidine, which can also be administered as second-line treatment of *T. b. rhodesiense*, has reported side effects that include hypotension, abnormalities of glucose metabolism, renal dysfunction, and gastro-intestinal symptoms [10,18]. The precise mechanism of action of pentamidine has not been determined. The drug enters the trypanosome using aquaglyceroporin 2 (TbAQP2) (see Figure 2 and references therein). Additional transporters involved are high affinity pentamidine transporters (HAPT1) and low affinity pentamidine transporters (LAPT1) encoded by TbAQP2 and TbAQP3, respectively. It is known that pentamidine interacts with trypanosomal kinetoplast DNA (kDNA), polyamine synthesis by decreasing the activity of ornithine, inhibition of RNA polymerase synthesis, and the synthesis of nucleic acid and proteins [19] (Figure 2). Pentamidine generates a cross-link between two adenines at 4–5 pairs apart in adenine–thymine-rich portions of *Trypanosoma* DNA. It also suppresses type II topoisomerase in the mitochondria of the parasites, culminating in a mitochondrial genome that is fragmented and unreadable [20]. Pentamidine is less toxic than suramin and is preferred against *T. b. gambiense*, whereas suramin is generally used against first-stage *T. b. rhodesiense* infections. Pentamidine resistance is associated with deletion, mutation, and rearrangements of the transporters. These transporters are also shared with melarsoprol. The drug resistance mechanism is selective and shows high levels of melarsoprol–pentamidine cross-resistance (MPXR) [21].

Suramin (Table 1) is one of the first anti-infective agents developed in medicinal chemistry programs and is still being used for the first hemolymphatic stage of infection (test dose 4–5 mg/kg body weight followed by 5 weekly doses of 20 mg/kg (<1 g) injected intravenously) [22,23]. Suramin sodium is a symmetric polyanionic sulfonated naphthylamine drug. It is likely to bind and inhibit various proteins. Thus, the many and different potential applications of suramin reflect its polypharmacology. Suramin enters the trypanosome through the endocytic pathway invariant surface glycoprotein ISG75 and a lysosome-based major facilitator superfamily (MSFT) (see Figure 2 and references therein). Suramin can inhibit numerous enzymes, namely *T. b.* glycolytic enzymes (hexokinase, aldolase, phosphoglycerate kinase, glycerol-3-phosphate dehydrogenase). The glycolytic enzymes are inside the glycosomes of the parasite, and it is still unclear how suramin could penetrate the membrane or if it could bind to glycolytic enzymes in the cytosol before their entrance in the glycosomes [24]. Alternative targets proposed for the trypanocidal effect of suramin are glycerophosphate oxidase, a serine oligopeptidase termed OP-Tb, the RNA-editing ligase REL1 of the trypanosome’s kinetoplast. It is unclear how suramin can cross the inner mitochondrial membrane; however, it was demonstrated that it is also able to inhibit oxidative phosphorylation in mitochondrial preparations of the trypanosomatid *Crithidia fasciculata* [25], supporting its capacity to cross the mitochondrial membrane. It inhibits cytokinesis in *T. b.,* as indicated by the finding that suramin treatment resulted in an increased number of trypanosomes with two nuclei. Treatment for the early stage of *T. b. rhodesiense* is with intravenous suramin (which can also be given as second-line treatment of *T. b. gambiense*), and, though effective, has the potential side effects of mild renal dysfunction, peripheral neuropathy, anaphylactic reactions, and bone marrow toxicity [18,25]. Drug resistance is associated with loss of functions of the internalization transporters [26].

### 3.2. Melarsoprol

Melarsoprol (Table 1) is a melaminophenyl arsenical derivative. To be effective in the second stage of the disease, antitrypanosomal drugs must cross the BBB. Pentamidine and suramin are not able to cross the BBB; therefore, they are only effective at reducing parasite infection during the first stage of disease, while the parasitemia is limited to the blood and lymphatic system. The treatment of late-stage *T. b. rhodesiense* is restricted to intravenous administration of melarsoprol. The drug administration is painful and extremely toxic. It shows a fatality rate of about 5–9% associated with a post-treatment reactive encephalopathy (PTRE) which is sometimes lethal. Peripheral neuropathy, skin rashes, cardiotoxicity, and agranulocytosis are among other side effects [27,28,29]. The current recommended dose is 2.2 mg/kg daily for 10 days, administered slowly by intravenous route, and is usually accompanied by prednisolone 1 mg/kg daily, orally. The mechanisms of action of melarsoprol and its drug resistance are complex. Melarsoprol is a prodrug, and it is converted into active melarsen oxide (Mel Ox). The activated form irreversibly binds the enzyme pyruvate kinase (PK) which is involved in the parasite’s aerobic metabolism of glucose. The PK inhibition by Mel Ox leads to alteration of the trypanosome ATP metabolism. In addition, Mel Ox interacts with trypanothione to generate the melarsen oxide–trypanothione complex (Mel T) and interacts with the spermidine–glutathione adduct that in the parasite replaces glutathione present in the human host. The melarsoprol metabolite reaches the cerebrospinal fluid, and concentrates in the trypanosomes, killing them. Unfortunately, melarsen and its metabolite interact with both the parasite and human proteins, thus explaining the observed adverse effect. Parasite resistance to melarsoprol has been associated with adenosine transporters, essential for melarsoprol transport into the parasite cytoplasm [30].

### 3.3. Eflornithine and NECT (Nifurtimox–Eflornithine Combination Therapy)

Eflornithine (Table 1) is an aliphatic carboxylic acid, (RS)-2,5-diamino-2-(difluoromethyl)pentanoic acid. It is prescribed for the treatment of the second stage of sleeping sickness associated with *T. b. gambiense*. The lower toxicity of eflornithine to the host with respect to melarsoprol has led to its use as the first-line treatment for HAT [31]. Melarsoprol is now second-line therapy for this variant. Eflornithine is effective in both the hemolymphatic CNS stages of West African Trypanosomiasis, and the second stage of *T. b. gambiense* infections, but less active against *T. b. rhodesiense*. The drug generates a dramatic reduction in symptoms and rapid clearing of parasites from blood and cerebrospinal fluid. For adults, the recommended dosage is 400 mg/kg per day in four divided doses for 14 days, and for children is 500 to 600 mg/kg per day on the same schedule. Eflornithine is an irreversible inhibitor of ornithine decarboxylase (ODC), which catalyzes the conversion of ornithine to putrescin and other polyamines that are important in cell division and differentiation [32]. The catalytic rate is high for the human enzymes with respect to the *T. b*. enzyme, showing rapid compound degradation. Therefore, its selectivity is based on the slower reaction in *T. b.* that induces a higher toxicity for the parasite. Its interference with polyamine synthesis impairs the parasite’s ability to maintain its redox state and neutralize reactive oxygen intermediates [32]. Eflornithine can be administered PO or IV and is well tolerated. Although it may also be administered alone as monotherapy, it is most commonly used in combination therapy with nifurtimox, the so-called NECT (nifurtimox-eflornithine combination therapy). Eflornithine monotherapy against *T. b. gambiense* is more toxic and less tolerated than NECT. NECT is less toxic than melarsoprol and is equally or more effective, and for several years it has been the initial therapy for *T. b. gambiense*. However, NECT is not effective against *T. b. rhodesiense* [33].

### 3.4. Nifurtimox (NFX)

Nifurtimox (NFX) (Table 1) belongs to the nitrofuran derivatives. It has been used against *T. cruzi*, which is the etiological agent of American Trypanosomiasis or Chagas disease. The drug lacks efficacy against *T. b.* when used as a single agent, therefore it is used in the treatment of the second stage of HAT as part of the NECT combination therapy. NECT is administer easier than NFX alone, and it reduces the eflornithine dosing regimen by 50%. NFX causes oxidative stress to the parasite [31]. It enters the trypanosome through the P2 transporter [34,35] (Figure 2). Its mechanism of action is based on cellular damage caused by a radical nitro anion derivative which is generated into a reduction cycle responsible for the formation of the superoxide anion and its reduction to the corresponding amine derivative. The selectivity of NFX for the parasite could be related to the presence of a nitro reductase (NTR) type I enzyme which is present in the parasite but not in mammals. This target selectivity could also be responsible for the lower side effects observed in mammals with respect to other drugs.

### 3.5. Fexinidazole

Fexinidazole (Table 1), a 5-nitroimidazole derivative bearing a methylthio function (SCH_3_), is the first all-oral treatment for HAT caused by *T.b. gambiense*, and it is safe and effective against both stages of the disease [36]. Due to its oral administration, it offers a series of advantages compared to NECT. Fexinidazole eliminates the need for IV drug treatment which requires skilled healthcare personnel and hospitalization. This in turn reduces the likelihood of infection, in addition to the overall drug burden that is imposed by eflornithine treatment [37].

Fexinidazole was developed as part of an innovative partnership between the Drugs for Neglected Diseases initiative (DNDi), the National Sleeping Sickness Programs of the Democratic Republic of Congo (DRC) and Central African Republic (CAR), and Sanofi. DNDi conducted a pivotal trial, which was a phase II and III randomized, non-inferiority trial (NCT01685827) that indicated that fexinidazole was both effective and safe in patients with late stage 2 HAT [36]. A subsequent prospective, multicenter, open-label, cohort study (NCT02169557) showed that fexinidazole is effective in patients with stage 1 or early stage 2 HAT [38]. Following the positive scientific opinion, granted in November 2018 by the European Medicines Agency, the drug was approved by the US Food and Drug Administration (FDA) on July 2021 as a first all-oral treatment for HAT.

In detail, fexinidazole was initially discovered back in the 1980s by Hoechst AG (now Sanofi) and was reclaimed following the joint effort of DNDi and the Swiss Tropical and Public Health Institute for the identification of new antitrypanosomal agents. In 2009, this molecule entered in phase I clinical trials [37,38,39] and moved to phase II and phase III trials in 2012 and 2017, respectively. A phase III trial evaluating fexinidazole in patients aged ≥6 years with Gambiense HAT of any stage treated either as inpatients or outpatients and including pregnant or lactating patients (NCT03025789) was initiated in November 2016 and was completed in March 2020. Finally, when the drug is administered properly, it is considered equipotent to pentamidine in the first stage of HAT and accordingly to NECT in the second stage. Currently, DNDi is the leading organization for the full access of patients to the drug and supports the corresponding pharmacovigilance study.

**Figure 3:**
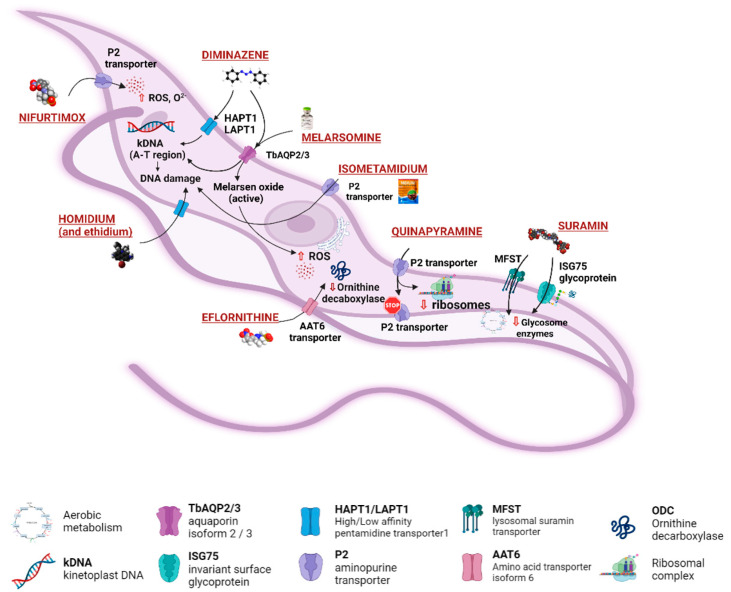
Drugs used to treat AAT and their mechanisms of action are illustrated. The different targets and transporters are reported [41,42,43,44,45,46,63,64,65]. Created with Biorender.com, agreement number SY23SM5AX6 13 April 2022.

Both nitro and SCH_3_ groups of fexinidazole are susceptible to metabolic reactions. The latter generate reactive amine species which are toxic and mutagenic to trypanosomes when interacting with electron chain transport [31]. Fexinidazole is active against *T. b. gambiense* and various other *T. b.* subspecies (including *T. b. rhodesiense* and *T. b. brucei*) in vitro and has demonstrated curative capacity in murine models of these trypanosomal infections, including acute infections with *T. b. gambiense* or *T. b. rhodesiense* and chronic infections with *T. b. brucei* [40]. In vitro studies carried out in *T. b. gambiense* have shown that fexinidazole and its two major metabolites, a sulfoxide (SOCH_3_, M1) and a sulfone (SO_2_CH_3_, M2), are responsible for its antitrypanosomal effects. Thus, it has been proposed that trypanosomes encode bacterial-like NTR that reduce fexinidazole and its M1/M2 derivatives to generate reactive metabolites that can induce kDNA damage to the trypanosome genome and its proteins [40] (Figure 3).

## 4. Current Treatments and Therapeutic Challenges for AAT

The World Health Organization (WHO) had set a target to eliminate HAT as a public health problem by 2020 [46]. However, this target has not been met due in part to the evolving epidemiological pattern of the disease, thus calling into question the fact that the Rhodesian form is zoonotic while humans are the main reservoirs for the Gambian form [47]. There has been slow progress in clinical isolates for HAT *gambiense* and HAT rhodesiense [48]. This situation is geographically specific (for African countries) and not widespread, demonstrating challenges to achieving the 2030 target. Domestic livestock infection with *Trypanosome* spp., namely small ruminants, is seldom screened in several developing countries, allowing these animals to continue acting as sources of sporadic infections in humans [5], raising the need to revise the current disease control strategy. In addition, the narrow chemotherapeutic spectrum available for AAT control will continue to undermine WHO control targets for HAT unless policy promotes innovations in the pharmaceutical industry. Trypanocidal drug applicability in the veterinary field ranges from prophylaxis to treatment of infected animals. Prophylactic administrations allow reducing the risk of infection in areas of intense tsetse activity and high risk of transmission, or when infected animals cannot be reached. This is the case in pastures inaccessible during monsoons, transhumant herds, or when livestock is on the move for trade in distant markets and forced to cross tsetse belts [49]. The treatment options (Table 2) include diminazene aceturate, isomethamidium chloride, and ethidium bromide (homidium salts), and accounted for 33%, 40%, and 26% of the veterinary prescriptions for AAT treatment [6,16]. However, diminazene and isometamidium are unable to penetrate the BBB, therefore are unsuccessful in the management of *T. b.*-related AAT. Suramin, the first line of treatment for HAT caused by *T. b. rhodesiense*, has been mainly used against *T. evansi* in camels and horses, but also in cases refractory to treatment [49]. Good prescription practices, also for chemoprophylaxis, include the application of curative trypanocides on sick animals alone and not for treatment of herds in blocks, as sub-therapeutic levels of these drugs contribute to the development of resistant parasite strains [49]. Systematic data on quantities and frequency of administration of trypanocides by sub-Saharan stakeholders in AAT management are missing as a consequence of the lack of surveillance in this topic. This challenges the quantification of the potential impact of treatment failure and drug resistance. To aggravate this scenario, it has been estimated that over 50% of the decisions regarding whether to treat AAT are often made by farmers. Trypanocides are being applied on animals based on unspecific health indicators and biased choices for drug selection. In the absence of veterinary consultancy towards a correct diagnosis and prescription, farmers purchase drugs directly from pharmacies and markets, operating without veterinary oversight, and consequently funding the proliferation of uncontrolled drug manufacturers and distributors. Unsupported clinical decisions often lead to the misuse and abuse of these drugs, namely the application of diminazene aceturate for Trypanosomiasis prevention when this drug lacks prophylactic capability or application of sub-optimal therapeutic dosages by inappropriate administration routes [50]. Ideally, identification of drug-resistant parasite strains would be performed by in vitro systems for parasite isolation and expansion from clinical isolation, followed by drug sensitivity assays. However, technical limitations have been found with *T. congolense*, *T. vivax*, and *T. evansi* (type B) testing in vitro due to difficulties in parasite isolation from clinical samples and consequent expansion in culture. To overcome these limitations, drug sensitivity can be assessed by parasitological ex vivo or in vivo assays. Ex vivo assays use blood from infected animals to perform short-time cultivation to evaluate the activity of selected drugs. Considering the short span of the parasite survival, radioactive incorporation of reporter molecules such as hypoxanthine is a useful technique to obtain an early readout of antiparasitic activity [51]. In vivo testing through inoculation of ruminants (reared in experimental settings) with blood collected from naturally infected animals can also be used. Animals are treated with a specific trypanocide drug and monitored for cure over a period of 100 days. Nevertheless, this approach is costly and difficult to manage, and it is unable to reflect field settings. Alternatively, mice can be inoculated with blood collected from a naturally infected animal and used for in vivo assays of drug availability and parasite resistance. Drawbacks of this method are related to the inability of some *T. vivax* strains to grow in mice. In these conditions, the results obtained in mice experiments are not always applicable in ruminants. Serum trypanocide drug concentrations can be determined by ELISA [52]. If the drug concentration in samples obtained from infected and treated animals is above a pre-defined cut-off (ex. 0.4 ng/mL for isometamidium), drug resistance is suspected [50]. From all the above, it is urgent to overcome the roadblock holding back the development of molecular tests seeking resistance-related genes among *Trypanosoma* spp. responsible for AAT. These tools are urgently necessary for feasible and cost-effective identification of drug-resistant parasites. The most used drugs to prevent and to treat AAT refer to four classes of compounds: phenanthridine, aminoquinalidine, diamidine, and melaminophenyl arsenical.

### 4.1. Phenanthridine (Homidium (or Ethidium Bromide), Isometamidium Chloride)

Homidium (or ethidium bromide) (Table 2) is a phenylphenanthridinium compound used in the management of AAT caused by *T. congolense* and *T. vivax* in large animals [53]. It was launched as an advancement over earlier phenanthridine-based trypanocidal agents, and it is also accessible as a chloride salt [54]. Treatment with homidium causes dyskinetoplasty that was also observed in the case of administration of other phenanthridines and diamidines. Genetic alterations are considered the general mechanism responsible for homidium trypanocidal effects. Homidium inhibits both kDNA and nuclear DNA replication in *T. b.* [55]. This is correlated with its flat aromatic structure that can induce a twisting and general alteration of the DNA double helix structure.

The isometamidium chloride formulations mainly include a mixture of four phenanthridine compounds, namely: isometamidium chloride hydrochloride [8-(3-mamidinophenyl- 2-triazeno)-3-amino-5-ethyl-6-chloride hydrochloride], [3-(3-m-amidinophenyl-2-phenylphenanthridinium chloride hydrochloride], the blue isomer [7-(mamidinophenyldiazo)-3,8-diamino-5-ethyl-6 phenylphenanthridinium chloride hydrochloride], and the disubstituted compound [3,8-di(3-m amidinophenyltriazeno)- 5-ethyl-6-phenylphenanthridinium chloride dihydrochloride]. Isomethamidium can bind the kDNA with high affinity with an unconventional sideways geometry. The drug is preferentially accumulated in the mitochondrion (gamma-subunit of ATPase). Mutation of the ATP synthase subunit is sufficient to cause a substantial level of resistance. It is mainly prescribed to treat early infections caused by *T. congolense* but is applicable to other *Trypanosoma* spp. [53]. It is administered by deep intramuscular injection, often causing lesions at the injection site. Isometamidium resistance has been associated with diminazene [50]. It is prescribed as curative in cattle at lower rates (0.25–0.5 mg/kg) and used as a prophylactic at 1 mg/kg. These drugs interacting with kDNA can also interact with human DNA and therefore can be potentially carcinogenic [49]. Isometamidium cannot pass the BBB, so its use is limited to treat first stage of infection. Recently, drug resistance was observed due to the alteration of the transport mechanism through the P2/TbAT1 transporter. This trypanocide is unsuccessful in the treatment of Trypanosomiasis caused by *T. b. evansi*, hence it is less widely utilized beyond sub-Saharan Africa.

### 4.2. Aminoquinaldine (Quinapyramine)

Quinapyramine (Table 2) is a conjugated bi-cyclic quinoline bearing a positive charge at neutral pH. This drug was first used in cattle to treat trypanosome infection until the 1970s, then it was stopped due to the increase in widespread resistance, and finally reintroduced again in 1984 to treat *T. b. evansi* in camels and horses [56]. The first prophylactic medication for AAT was a combination of soluble sulphate and insoluble chloride salts of the drug. The therapeutic regimen of 4–7 mg/kg of the drug showed either a prophylactic or curative effect when used for four continuative months. Quinapyramine is unable to cross the BBB, and the liver and the kidneys represent the main accumulation sites, where the high drug levels remain elevated for weeks and can cause organ-specific toxicity. The specific mechanism of action and resistance against the parasite remain unknown. It is reported that quinapyramine can interfere with nucleic acid synthesis, cytoplasmatic ribosome inhibition (Figure 3), changes in the potential of mitochondrial membranes, and interference with the entry of the trypanocides into the mitochondria itself [57].

### 4.3. Diamidine (Diminazene)

Diminazene (Table 2) is an aromatic diamidine consisting of two amidinophenyl moieties linked by a triazene bridge. It is marketed as a diaceturate salt. It is the most widely used therapeutic agent for Trypanosomiasis in domestic livestock, as it has a higher therapeutic index in most livestock species compared to other trypanocides. It shows a low rate of acquired resistance, but some strains of *T. congolense* and *T. vivax* could have developed resistance mechanisms as a consequence of bad therapy management [53]. Due to its activity against both *T. congolense* and *T. vivax*, it is the most used trypanocide in cattle, sheep, and goats infections. It is a curative agent, not a prophylactic one, and it is rapidly metabolized and excreted. The treatment consists in a dose of 3.5 mg/Kg for *T. congolense* and *T. vivax* infections and a dose of 7 mg/Kg for *T. brucei* infections administrated intramuscularly or subcutaneously. The higher value for *T. brucei* is probably due to its wider tissue distribution. Diminazene enters the trypanosome through the TbAT1/P2 transporters (Figure 3). It can bind the minor groove of DNA in AT-rich sites, causing replication inhibition and kDNA loss, which may be exacerbated by an inhibitory effect on mitochondrial type II topoisomerase (Figure 3) [58]. The compound interferes with the potential of the mitochondrial membrane. It alters the host immune response by reducing pro-inflammatory cytokines and excessive immunological activation. Diminazene uptake needs P2 and TbAQP2/3 transporters to cross cell membranes. It cannot cross the BBB and therefore it is not useful in the second stage of the infection. Diminazene is prescribed for HAT treatment despite its high toxicity. Drug resistance occurs due to loss of P2 transporter efficacy encoded by the gene TbAT1, as well as deletion and rearrangements of TbAQP2/3 genes.

### 4.4. Melaminophenyl Arsenical

Melarsomine dihydrochloride (Table 2) is a bis (aminoethylthio) 4-melamino- phenylarsine dihydrochloride showing a different profile with respect to melarsoprol. The melarsen oxide, its active metabolite, penetrates *T. b.* cells via the P2/ TbAT1 adenosine nucleoside transporter and TbAQP2 transporters (Figura 3). The mechanism of action of arsenical drugs includes alterations in glucose uptake and metabolism, inhibition of glutathione reductase, and alterations of the structure and function of the parasite intestinal epithelium [59]. Administered at a dosage of 0.25 and 0.5 mg/kg, it has been successful in horses with acute and chronic dourine. Melarsomine shows low selectivity for the parasite and significant toxicity. The most important target identified is the parasitic adenosine nucleotide transporter; therefore, the specificity of the treatment for AAT with respect to HAT should rely on different schedules or pharmaceutical approaches and monitoring of drug resistance to preserve the drug efficacy [60].

### 4.5. Suramin

Suramin is the oldest trypanocide still in routine veterinary use, introduced in 1921 against “surra” in camels and now employed against “surra” or equine Trypanosomiasis by *T. b. evansi*, because it is more effective than diminazene and less toxic than quinapyramine. The treatment requires 10 mg/Kg intravenously; intramuscular administration is avoided because it causes intense local irritation. It is a curative and prophylactic drug active primarily against *T. b. gambiense* and *T. b. rhodesiense.* If complexed with antrycide dimethyl sulphate, suramin is also active against *T. vivax* and *T. congolense* [22]. Suramin is not able to cross the BBB, but it binds to circulating proteins and cytosolic trypanosome enzymes by electrostatic interaction. Its mechanism of action has not been determined yet, but it is suggested to decrease the glycolytic ATP production in *T. brucei*, by inhibiting glycerol-3-phospate dehydrogenase (GDPH) of the mitochondrial phosphorylation pathway (Figure 3). Many targets were proposed, including the enzymes 6-phosphogluconate dehydrogenase, other enzymes of the pentose phosphate pathway, and Ca^2+^ channels. They can be involved in the mechanism of parasite death. The inhibiting uptake of suramin is sufficient to induce parasite resistance to the drug [61,62].

## 5. Recent Clinical Candidate Drugs for HAT

Over the years, HAT clinical therapies have relied on the administration of drugs associated with severe side effects, toxicity, difficult administration, low availability, and high cost [8]. The ideal drug against *Trypanosoma* spp. infections should be possibly active against all parasite subspecies and effective in disease stages. It should have low toxicity compared to the available treatment options and it should be suitable for oral administration to achieve better availability and increase treatment effectiveness. These limitations have prompted the search for alternative active substances (such as those of natural origin) for the management of HAT [53]. The pipeline of clinical candidates is very limited. As reported above, the most important drug introduced in therapy recently is fexinidazole. This oral drug, showing equal efficacy and safety, could have replaced NECT. A phase III trial including a vulnerable patient population initiated in November 2016 and completed in March 2020 did not disclose the outcome of the study [66]. Only two other candidates are described, parafuramidine and acoziborole, which show a different profile and outcome.

### 5.1. Parafuramidine

DB289 (Table 3), the methoxy product of furamidine (2,5-bis(4-amidinophenyl)-furan) in which the benzamidine moieties are linked through a furan ring, emerged as the lead compound for progression towards clinical studies. Furamidine is highly polar and unable to cross lipid bilayers without transporters. Parafuramidine, however, its N-oxime derivative, has much greater capacity to diffuse across membranes, including the intestinal epithelium, providing considerable oral bioavailability [67]. Once systemic, it is metabolized by various cytochrome P450 enzymes and cytochrome b5 reductase to furamidine (DB75) [68]. The drug was first employed in HIV treatments as an orphan drug (FDA) and reached clinical trials as an antiparasitic agent. Assessments were halted in 2008 due to the appearance of renal toxicity during extended phase I safety profile studies. The aza-derivatives of DB75, including DB868, a prodrug of DB829, and DB844, a prodrug of DB820, also showed activity against stage 2 disease, but development was interrupted after the identification of the toxicity associated with parafuramidine metabolites [69]. Despite the fact that the development was interrupted, the toxicity can be modulated with additional medicinal chemistry elaboration, and some studies are ongoing in this direction. Most likely, the development of the core diamidine scaffold will continue towards active and less toxic compounds.

### 5.2. Acoziborole

Acoziborole (SCYX-7158) is used as a single dose oral cure for stage 2 HAT. Benzoxaboroles are a class of molecules characterized by a core scaffold based on an oxaborole heterocycle fused to a benzene ring. Activity against trypanosomes was first reported in 2010, after the scaffold entered preclinical studies, and DNDi (the Drugs for Neglected Disease initiative) selected this class for further work. One of the impressive properties of this compound is its capacity to reach the CNS and retain trypanocide activity levels long enough to obtain a cure. Recent studies involving the development of a *T. b. brucei* overexpression library with optimized genome coverage followed by a biological screening enabled the identification of the parasite mRNA processing endonuclease, and that cleavage and polyadenylation specificity factor 3 (CPSF3) is the target of the promising new class of benzoxaboroles [68]. Specifically, acoziborole (AN5568) is in clinical trials for HAT and AN11736 for Nagana (for the treatment of AAT in early development studies). Thus, it was shown that the trypanocidal activities of acoziborole (for HAT) and veterinary AN11736 are attributed to the perturbation of the polyadenylation and trans-splicing activities enabled by the trypanosomal enzyme CPSF3. The mentioned target was identified through a wide omics study performed on a genome-scale gain-of-function library developed in trypanosomes [69]. Furthermore, metabolomic studies pointed to the perturbation of S-adenosyl-L-methionine metabolism [70]. Its modest in vitro potency against *T. b*. (IC_50_ around 0.6 µM) was offset by good in vivo pharmacokinetic properties, giving a 100% cure in a mouse model of stage 2 disease following an oral dosing of 25 mg/kg once a day for seven days [24]. Preclinical testing showed acceptable toxicity in mice or dogs with a concentration of no observed adverse event limit (NOAEL) of 15 mg/kg. No modulation of key proteins (CYP450), serine/cysteine peptidase, or hERG channels emerged. Acoziborole was also non-mutagenic in the Ames tests or standard mammalian cell genotoxicity assays [71]. Hence, phase I clinical safety trials of acoziborole were developed in 2012 and became the first new chemical entity resulting from DNDi’s program to enter clinical trials for HAT. A phase I study that included 128 healthy male subjects of sub-Saharan African origin was conducted in 2015 in France to assess safety, tolerability, pharmacokinetics, and pharmacodynamics after single oral, ascending doses [18]. Some adverse effects were noted but not severe, and the trial led to the selection of the 960 mg dose, given as a single administration in three tablets (320 mg each). Outputs from the phase I study were satisfactory to start the phase II/III trials in Africa in 2016 [72]. Early indications (unpublished) suggest remarkable efficacy and safety profiles.

## 6. Recent Pharmaceutical Discovery and Development against African Trypanosomiasis

The search for new drug candidates with application in the field of parasitology is still very difficult compared to other diseases. The reasons are to be found in the scarcity of dedicated research funds and the lack of interest on the part of pharmaceutical giants to invest in the face of limited revenues. However, several research groups and institutions are actively working to try to meet this need. The current focus of research is the development of new hits/leads towards biological targets divergent from those present in eukaryotic host cells. This approach should reduce toxicity by favoring species specificity. Below, we report some of the most recent and promising hits/leads for the development of new, more effective, and safer drugs for the treatment of HAT and AAT.

### 6.1. Nucleoside Analogues

Protozoan parasites, in contrast with their mammalian hosts, are not able to synthesize the essential purine nucleosides de novo for their survival. Hence, these parasites have developed a set of purine salvage enzymes that facilitate the acquisition of purines from their host [73,74]. This observation renders the synthesis of purine nucleoside analogues as potential antitrypanosomal agents a very appealing strategy. Indeed, this approach has yielded a series of very promising compounds such as tubercidin [75], cordycepin [76,77], and formycin B [78] (Table 4).

Tubercidin is a nucleoside antibiotic with very good antiparasitic activity; however, it is highly toxic to mammalian cells. In an effort to improve the activity and reduce the toxicity, Hulpia et al. synthesized and tested in vitro a series of modified tubercidin analogues bearing phenyl groups of various electron density at the C-7 position [79]. Finally, the replacement of the phenyl ring by a pyridine ring led to compound **1,** which showed nanomolar potency in vitro and reduced toxicity, but it was also accompanied by poor metabolic stability. The same research group expanded their studies by combining the scaffolds of tubercidin and cordycepin. These hybrids showed even better antitrypanosomal activity, and especially analogue **2** is considered a very potent and orally bioavailable lead compound [80]. In general, nucleoside analogues can offer an additional advantage in terms of biological activity. Analogue **2** can penetrate the BBB and thus it can be more potent for the treatment of late-stage HAT.

### 6.2. New Molecules from Structure-Based Drug Design (SBDD)

Blaazar et al. reported a series of tetrahydrophthalazinone derivatives as inhibitors of the *T. b.* 3′,5′-cyclic nucleotide phosphodiesterases B1 (TbrPDEB1), cyclic AMP-specific PDEs, providing useful structure–activity relationship (SAR) studies for further optimization of parasitic PDE-specific inhibitors [81]. Virtual screening and structure-based design led to the identification of novel diarylether substituted tetrahydrophthalazinones as TbPDEB1 enzyme inhibitors with an IC50 lower than 1 µM in vitro against T. *bi* parasites and selectivity more than 30-fold with respect to the human PDE4 (Table 4) [81]. Poehner et al., taking advantage of a fragment-based drug design approach, synthetic elaboration, and crystallographic structure determination, reported a series of pteridine derivatives showing a sub-nanomolar inhibition constant (Ki) of *T. brucei* pteridine reductase 1 (TbPTR1), selective inhibition of Tb dihydrofolate reductase (TbDHFR) over human dihydrofolate reductase (hDHFR), and micromolar EC50 against *T. b.* (Table 4). This work provides the basis for the optimization of this series of compounds to be developed for in vivo studies [82]. Furthermore, after the discovery of a new first series of pyrazole sulfonamide able to inhibit *T.b.* N-myristoil transferase (TbNMT), this ubiquitarian parasitic enzyme was validated as a promising target for drug discovery with a co-crystallization between DDD85646 and NMT [83]. An additional ligand-based drug design from Spinks et al. led to the discovery of two new scaffolds able to target TbNMT, 2,3-arysubstituted thiazolidinones and N-alkyl benzomorpholinone, both accommodating in the same allosteric pocket as DDD85646 [84]. The most active hit of the former class has an IC50 of 22 uM on TbNMT, but a comparable selectivity index on the human enzyme isoform. On the other hand, N-alkyl benzomorpholinones target the parasite enzyme in a more specific manner (IC50 on hNMT > 100 uM) and demonstrate a low-micromolar IC50 if the core structure is properly functionalized. Furthermore, a combination of a methyl-isoxazole on the benzomorpholinones nitrogen and a long alkyl chain on the benzyl ring, terminating with a cyclic tertiary amine, allowed to achieve a nanomolar potency (IC50 < 2 nM, EC50 < 10 nM). This work laid the foundation for the synthesis of selective lead compounds with potent antiparasitic effects ready for a stage 2 in vivo efficacy study [84] (Table 4).

### 6.3. Chemotypes Derived from High-Throughput Screening (HTS)

Benzoxazepinoindazoles are a class of compounds which emerged as potent antitrypanosomal agents upon a HTS of human kinase inhibitors against *T. brucei*. After extensive SAR studies, compound **3** showed excellent activity, a favorable pharmacokinetic (PK) profile, and very good selectivity [85]. Thus, its activity was further evaluated in vivo. Compound **3** was able to cure the 60% of mice in a systemic model of HAT, and it can successfully cross the BBB. However, it was unable to clear parasitemia in a CNS model of the disease. Following the same approach, diaminopurines were also identified as compounds with antitrypanosomal activity. Optimization of hit-compounds led to a new promising entity with improved absorption, distribution, metabolism and excretion (ADME) properties (compound **4**, Table 4) [86]. Compound 7-azaindole, is another scaffold that emerged as a potent growth inhibitor of *T. b*. From a screening study of more than 40,000 kinase inhibitors, 797 hits were obtained for further evaluation [85]. Among these hits, a series of 3,5-disubstituted-7-azindole compounds was found the most potent and developed in a hit-to-lead optimization process. Analogue **5** was identified as a lead compound, exhibiting sub-micromolar potency against *T. b.* accompanied by very good aqueous solubility and high in vitro human liver microsome intrinsic clearance. Compound **5** was qualified for in vivo PK studies, but unfortunately it showed low BBB penetration which is essential for the treatment of stage 2 HAT.

## 7. Drug Resistance in *T. brucei*

Drug resistance in *Trypanosoma* is associated with genetic modifications that lead to changes in uptake, efflux, metabolism, and target interactions (Figure 2 and Figure 3). This leads to a decrease in therapeutic potential of the drugs by an increase in the effective concentration required for achieving the trypanocidal concentrations [56]. The development of drug resistance is associated with continuous exposure to sub-lethal concentrations of drugs. The peculiarities of the drug used against AAT (and HAT to a lesser extent) potentiate resistance development due to the existence of significant periods of the mentioned sub-lethal drug concentrations in the body. These periods are potentiated by unrestricted large-scale use of the drug, inaccurate dosing regimens, and use of drugs that are slowly eliminated from the body. Drug-specific selection mechanisms normally follow the primary low impact generic resistance mechanisms associated with increased stress responses [87]. When drug-specific resistance events appear, the increase in the drug concentration will only lead to a greater selective pressure resulting in higher levels of resistance. Data on AAT demonstrate that about a decade of use of antimicrobials is sufficient to develop drug resistance [88]. The most commonly known mechanisms of resistance in trypanosomes are associated with impaired cross-membrane transport [60]. Isometamidium resistance is potentiated by reduced immunity and is mostly associated with the decreased uptake of the drug through still unknown mechanisms [89]. P2 aminopurine transporters encoded by TbAT1 are associated with nifurtimox and diamidine (pentamidine and diminazene) resistance. Loss of function of the invariant surface glycoprotein ISG75 is associated with suramin resistance. The P2-purine transporter is associated with resistance to melarsoprol and diamidines (pentamidine and diminazene). The aminoacidic transporter AAT6 is associated with eflornithine resistance. Aquaglyceroporin 2 is associated with pentamidine and diminazene resistance [60]. Efflux mechanisms such as the overexpression of P-glycoprotein efflux pumps was also associated with melarsoprol resistance [90]. Resistance to isometamidium might also be associated with active extrusion [91]. Non-transport-related drug resistance mechanisms can be attributed to mutation in the F1Fo-ATO synthetase in dysninetoplastic *T. b.* This enzyme is crucial in cellular energy interconversion. A proton gradient is necessary during the process of the ATP synthesis to lead to significant resistance to isometamidium and homidium [92]. Moreover, due to the development of resistant strains infecting both humans and animals, it can be the cause of cross-resistance among different species other than within the same species. In HAT management, drug-resistant *T. b. rhodesiense* has a devastating impact on public health and compromises the control measures for this parasite. Melarsoprol resistance and safety issues have led to 30% failure in HAT treatment. Resistance to pentamidine has been reported, and more seriously, cross-resistance between melarsoprol and pentamidine [17,37]. In addition to acquired drug resistance mechanisms, *T. b. rhodesiense* appears to be naturally refractory to eflornithine. For AAT, the extensive use of the limited drug option available is normally associated with overuse and under-dosage, which has resulted in the establishment of drug resistance and even more seriously to the advent of cross-resistance. The latest information from Africa reports endemic drug resistance in 21 African nations [17].

Experimental studies on the effectiveness of ivermectin on *T. b. brucei* are being carried out, aiming to find a therapeutical option to replace suramin, since ivermectin is less toxic and orally bioavailable. Ivermectin is a semi-synthetic macrolide with effective broad-spectrum antiparasitic activity. It is highly efficacious, acting robustly at low doses against a wide variety of nematode, insect, and acarine parasites. Despite being registered for human use since 1987, ivermectin’s mechanisms of action are yet to be unveiled [10]. Ivermectin-resistant parasites appeared in treated animals, as well as in ectoparasites, such as copepods parasitizing salmon in fish granges, but drug resistance was not observed in the parasite infecting the human populations, at least in those patients who have been taking ivermectin as a monotherapy for over 30 years [21].

## 8. Surveillance and Disease Control Measures: Targets for African Trypanosomiasis Elimination under One Health Umbrella

An effective and sustainable control of African Trypanosomiasis will be directly reflected on agricultural development and veterinary and public health, therefore contributing to poverty alleviation and population wellbeing. Animal health, public health, and economic needs sustain the urge to control Trypanosomiasis in sub-Saharan Africa. Since treatment of AAT reduces the animal reservoirs of zoonotic vector-borne *Trypanosoma* spp., One Health programs must serve as foundations for HAT elimination. Therefore, all the players involved in the epidemiologic scenario must be considered in the HAT elimination programs [93]. As a clear indication of the correlation between HAT and AAT infections, it was shown that trypanocidal treatment of cattle decreases the human cases due to *T. rhodesiense* [94]. Control of parasite transmission is the key point for HAT and AAT management. Since multi-factorial conditions participate in the life cycle of this arthropod-borne zoonotic parasitic disease, disease prevention and treatment must consider the pathogenicity of the infecting *Trypanosome* spp., infection of animal hosts, vectors of transmission, human infection, and the environment. In this context, responsible and long-lasting actions must be thoughtfully and scientifically drawn under the One Health framework to protect the health and integrity of wildlife, environment, animal, and human health, under transparent interdisciplinary policies. Good examples of effective disease control policies come from Togo and Côte d’Ivoire, who eliminated HAT as a public health problem since 2020. The reduction in the incidence of human infection in endemic areas, limiting the burden of the human reservoir, resulted from an active case detection in villages at risk and vector control. Patients presenting clinical signs of illness had a diagnosis supported by laboratory tests, allowing an accurate case detection and the implementation of adequate curative treatments. Upon control of the disease incidence, surveillance sentinel sites were implemented in the hospitals covering the main areas exposed to HAT [95]. The inability to diagnose cases of subclinical infection and asymptomatic carriers raises concerns about the possibility to control the infection and must be addressed. Uganda is two steps away from eliminating *T. b. gambiense* as a public health hazard after decades of epidemics complicated by civil war. Over the last decade, confirmed cases of sleeping sickness have sharply declined thanks to Uganda’s Ministry of Health Trypanosomiasis Control Council (UTCC) and the efforts of international partners to engage other ministries and the community in the implementation of control strategies. The success of this strategy is largely attributed to the engagement of stakeholders and strategies including the use of insecticides to treat cattle as live bait for tsetse, therefore breaking the transmission of trypanosomes to both humans and animals [96]. This fine balance is at risk of disruption with the influx of refugees from South Sudan, because in that country the transmission of *T. b. gambiense* is still a problem [97].

Resistance to trypanocidals is becoming common, cross-resistance between classes has been identified, and resistant trypanosomes may pass from animals to humans. Since human–animal proximity increases the risk of spill over and crossing species barriers, the interchange of *Trypanosoma* spp. between animals and humans must be carefully monitored [71]. Part of the prevention measures to break the transmission pathway of drug-resistant strains is to limit the application of specific antiprotozoals in animals, independently from drugs to treat HAT.

Regarding vector control measures, both chemical (e.g., spraying insecticides on the environment and animals) and biological interventions (e.g., deforestation and destruction of tsetse natural habitats, trapping flies, wild animal elimination) and genetic control of these flies (releasing sterile insects into the wild) have been attempted. The interventions did not give effective results, and caused severe environmental consequences, besides the selection of insecticide-resistant tsetse flies [6]. At the moment, there are no reliable and environmentally safe options for tsetse control. Control measures targeting AAT have been based on chemoprophylaxis, which is most of the time inaccessible to resource-poor smallholder livestock keepers. Devastating consequences have surged over the last decades from the misuse and abuse of veterinary trypanocidal agents in animals. This common practice caused the rise of drug-resistant parasites [49]. Certain African livestock breeds present an immune-tolerant phenotype towards some *Trypanosome* species, for which the underlying mechanisms are yet to be discovered. Genetic combinations among different breeds and the introduction of trypanotolerant phenotypes may result in a balance between productivity and resistance to Trypanosomiasis, leading to a sustainable production and economical benefit [6]. From the above, programs aiming at the interruption of the zoonotic *Trypanosoma* spp. life cycle must prioritize collaboration and coordination of efforts at national, provincial, and district levels to integrate human health and veterinary and agricultural sectors together with wildlife and natural resources advocates, epidemiologists, policy makers, researchers, the pharmaceutical industry, and all those involved in epidemic preparedness. A framework must be designed in order to achieve: (i) depletion of parasite reservoirs through active case detection (serological screening) and treatment of the infected humans and domestic animals; (ii) ensure the best available treatment is employed and is adequate for the disease stage; (iii) reduction in the tsetse fly population and human and animal contact with tsetse flies by protecting population health vulnerabilities; (iv) reduction in human–wildlife and livestock–wildlife contact; (v) detection of both human and animal cases; (vi) development of effective communication strategies between all intervenors and implementation of guidelines and training opportunities for health workers; (vii) translation of research findings to solutions in order to support the development of new safer drugs for HAT and AAT prevention, more advanced treatments, and field-friendly techniques for an early diagnosis [4]; and (viii) development of new drugs with appropriate profiles. The overall impact of the drug manufacturing and waste from hospital and livestock management should be considered and an appropriate effort to reduce the impact of all waste water on the environment is needed. One should consider a separate drug application on humans and animals. In particular, careful consideration about which targets to select for the drug discovery programs, protein sequences, and genetic studies is needed, to understand drug resistance development for the specific target and susceptibility to mutations. An appropriate target product profile should be designed to achieve a drug that is low cost, easy to manage, chemically stable, and selective for a target population. This would prevent rapid drug resistance development and can be effective in case of parasite transmission.

## 9. Conclusions

Both AAT and HAT control is deeply asymmetric among sub-Saharan African countries. Multiple factors and diverse reasons are responsible for different epidemiologic scenarios. However, geopolitical conflicts, drought, agricultural massification, deforestation and human invasion of tsetse endemic sites, lack of sensitive diagnostic tools adequate for field work and detection of subclinical infection, lack of control of AAT, reduced therapeutic options, and development of trypanosome drug-resistant strains, combined with nonexistent or poorly designed surveillance strategies that are often not supported by One Health principles and frameworks, leads to poor disease epidemics.

Re-emergence of HAT should be prevented by an integrated approach including: (i) an effective surveillance of infection among wildlife, domestic animals, and humans; (ii) implementation of a vector surveillance system and control measures; (iii) implementation of an effective system for disease notification in both animals and humans; (iv) implementation of effective, sensitive, and fast diagnostic tools applicable to field conditions; (v) creation of an effective network linking communication between stakeholders, medical and veterinary departments, and government and policy makers; and, finally, investment in the development of safer and more easily available HAT and AAT therapies and prophylactics. Cooperation among different actors will make this approach feasible and sustainable.

## Figures and Tables

**Table 1:** Drugs in current treatment for Human African Trypanosomiasis (HAT).

Approved Drugs for HAT	Species	Dose
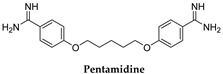	*T. b. gambiense*	4 mg/kg/day IM × 10 days
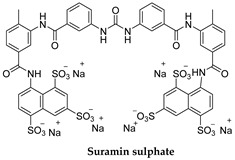	*T. b. rhodesiense* *T. b. gambiense*	Test dose of 4–5 mg/kg (day 1), then 20 mg/kg, weekly ×5 weeks (maximal dose/injection: 1 g)
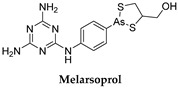	*T. b. rhodesiense*	2.2 mg/kg/day × 10 days, usually accompanied by prednisolone1 mg/kg/day
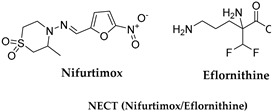	*T. b. gambiense*	Nifurtimox: 15 mg/kg 24 h, 3 dose × 10 days Eflornithine: 400 mg/kg/day 2-h infusion × 7 days
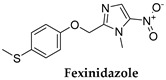	*T. b. gambiense*	For patients 20–35 kg; 1200 mg/day × 4 days and then 600 mg/day × 6 days For patients ≥ 35 kg; 1800 mg/day × 4 days and then 1200 mg/day × 6 days

**Table 2:** Drugs in current treatment for Animal African Trypanosomiasis (AAT).

Drugs in Current Treatment for AAT	Trypanosome Species Target	Animal Species	Applicability
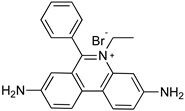	*T. vivax* *T. congolense* *T. b.*	Cattle Sheep Goats	Treatment
Homidium bromide			
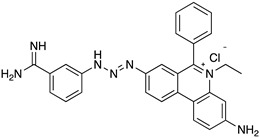	*T. vivax* *T. congolense* *T. b.*	Cattle Sheep Goats Horses	Treatment or prophylaxis
Isometamidium chloride			
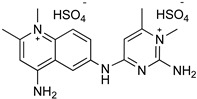	*T. b.* *T. evansi*	Horses Camels Cattle	Prophylaxis
Quinapyramine sulphate			
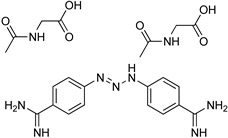	*T. congolense* *T. evansi*	Cattle Sheep Goats Dogs	Treatment
Diminazene aceturate			
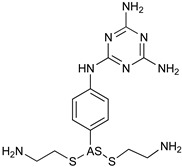	*T. evansi*	Camels	Treatment
Melarsomine			

**Table 3:** Parafuramidine and aza-derivatives in which the colored atoms indicate the structural changes with respect to the drug of reference.

Parafuramidine, Furamidine and Aza-Derivatives	
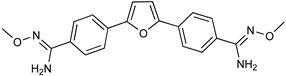	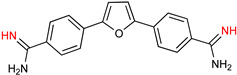
Parafuramidine (DB289)	Furamidine (DB75)
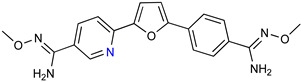	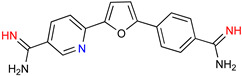
DB844	DB20
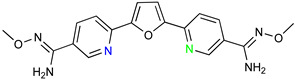	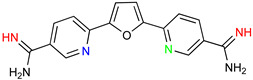
DB868	DB829

**Table 4:** Recent pharmaceutical discoveries against African Trypanosomiasis (HAT and AAT).

Recent Pharmaceutical Discovery for the Treatment of HAT and AAT		
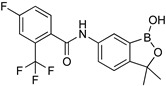	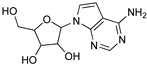	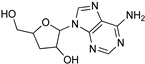
Acoziborole (SCYX-7158)	Tubercidin	Cordycepin
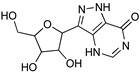	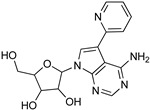	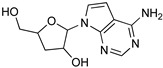
Formycin B	1	2
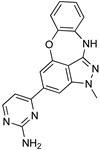	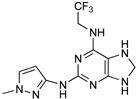	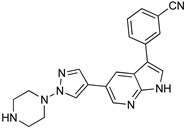
3	4	5
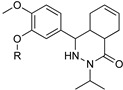	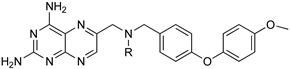	
Diaryl ether substituted tetrahydrophthalazinones scaffold	Pteridine derivatives scaffold	
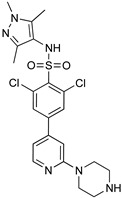	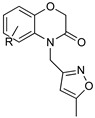	
DDD85646	Benzomorpholinone scaffold	
